# Experience with hip denervation in non-operative hip fracture care for frail older patients in the Netherlands: an interview study

**DOI:** 10.1136/bmjopen-2024-095738

**Published:** 2025-05-08

**Authors:** Thamar Kroes, Johanna M van Breugel, Rachel Smits, Henk Jan Schuijt, Detlef Van der Velde, Hanna C Willems, On behalf of the Dutch PENG Study Group, Renée A G Brüggemann

**Affiliations:** 1 Maastricht University Medical Center, Maastricht, Netherlands; 2 University Medical Center Groningen, Groningen, Netherlands; 3 Elizabeth Twee Steden Ziekenhuis, Tilburg, Netherlands; 4 Rijnstate, Arnhem, Netherlands; 5 St. Antonius, Utrecht, Netherlands; 6 Zuyderland, Heerlen, Netherlands; 7 Radboud University Medical Center, Nijmegen, Netherlands; 8 Erasmus Medical Center, Rotterdam, Netherlands; 9 OLVG, Amsterdam, Netherlands; 1 Department of Internal Medicine, Section Geriatrics, Amsterdam UMC Locatie AMC, Amsterdam, The Netherlands; 2 Department of Trauma Surgery, St Antonius Hospital, Utrecht, The Netherlands; 3 Department of Anesthesiology, Pain and Palliative Medicine, Radboud University Medical Center, Nijmegen, The Netherlands

**Keywords:** Pain management, Ultrasonography, PALLIATIVE CARE, Trauma management, Frail Elderly, QUALITATIVE RESEARCH

## Abstract

**Abstract:**

**Objective:**

The objective was to explore treatment experience of hip denervation via PEricapsular Nerve Group block with phenol in non-operative management and end-of-life (EOL) care after hip fractures.

**Design:**

A qualitative study was conducted with semistructured interviews. The interviews were analysed using thematic discourse analysis.

**Setting and participants:**

The study was conducted in a large regional hospital in the Netherlands. Proxies (first-contact person, often a first-degree or second-degree relative) of frail older adults treated between January 2022 and June 2023 were included, as patients had either cognitive impairment or were deceased.

**Results:**

The process surrounding hip denervation was emotionally charged due to the EOL setting and preceding discussion on whether or not to operate. The EOL setting impaired information uptake in participants and complicated communication. Hip denervation was experienced as a partial source of comfort. Logistics and aftercare were described as suboptimal. Participants emphasised the importance of a dignified and autonomous EOL phase.

**Conclusions:**

This study describes treatment experience from the patient–proxy perspective. It highlights the importance of a provider setting attuned to EOL care needs. Adequate pain management, effective communication and realistic autonomy for patients and proxies are warranted.

**STRENGTHS AND LIMITATIONS OF THIS STUDY**

- Use of transparent and well-regarded qualitative methods.
- Including cognitively impaired patients.
- Patient–proxy perspectives may introduce proxy bias.
- The national scope limits applicability in diverse cultural contexts.

## Introduction

 Due to the ageing population, hip fractures in frail older patients pose a growing healthcare concern.^1 2^ Frailty in hip fracture patients changes the balance between the burden of operative management (OM) and revalidation versus expected postoperative recovery.^3^,^5^ Non-operative management (NOM) for hip fractures is considered a viable alternative for selected frail older patients in end-of-life (EOL) care.^6^ NOM involves consideration of prognosis and patient preferences in shared decision-making with patients and their proxies.^7^,^9^ Proxies are closely involved due to the high prevalence of cognitive impairment in this population.^10^,^13^

NOM is aimed to align with the three essential components of EOL care: whole-person care, mortality acknowledgement and focus on quality of life.^14 15^ Effective pain management is the most critical aspect in NOM, alongside early EOL care consultation and patient–proxy interaction.^716^,^18^

Pain management in NOM was traditionally achieved by systemic analgesics with often undesirable side effects, such as delirium and loss of consciousness.^19 20^ Currently, local pain management techniques, such as the PEricapsular Nerve Group (PENG) block, are available for selected frail older patients receiving NOM.^21^ The PENG block is an ultrasound-guided block of the anterior hip capsule.^22 23^ It targets the sensory articular branches of the femoral nerve, the obturator nerve and, when present, the accessory obturator nerve, resulting in pain relief with preservation of the motor function.^22 24^ Given this targeted approach, chemical hip denervation with phenol or ethanol is possible for long-lasting pain relief and is implemented in NOM for frail older hip fracture patients.^21 25^ Hip denervation is offered directly after the decision to opt for NOM, with the aim of providing pain relief until the decease.

Decreasing care-related pain and reducing the need for systemic opioids through hip denervation are hypothesised to improve treatment experience. However, research on treatment experience is currently lacking. This study aims to explore treatment experience regarding hip denervation with phenol via PENG block in an EOL setting.

## Methods

### Study design

An exploratory interview study was conducted in a large regional hospital in the Netherlands between 1 May 2023 and 1 August 2023. All adults aged 70 years and older, diagnosed with a femoral neck or intertrochanteric hip fracture, treated with NOM and hip denervation with phenol via PENG block in an EOL setting were eligible for inclusion. Patients were included within a predetermined time frame based on hospital admission date (1 July 2022 until 30 June 2023). Hip denervation was preceded by a shared decision-making process on NOM. Afterwards, the anaesthesiologist was consulted on the indication and feasibility of hip denervation. Patients all received 10 mL of 6% phenol via an ultrasound-guided PENG block. The full clinical treatment protocol is available in online supplemental appendix A.

Study participants were restricted to proxies because patients were either cognitively impaired or deceased. A proxy was defined as the first contact person or a first-degree or second-degree relative of the patient. This study was conducted from the patient–proxy perspective, which entails that proxies were asked ‘to assess the patient as they think the patient would respond’.^26^ Studying patient experiences through proxies is appropriate according to the constructivist perspective in which experiences are socially produced and reproduced, rather than inherent to individuals.^27 28^

**Figure 1:**
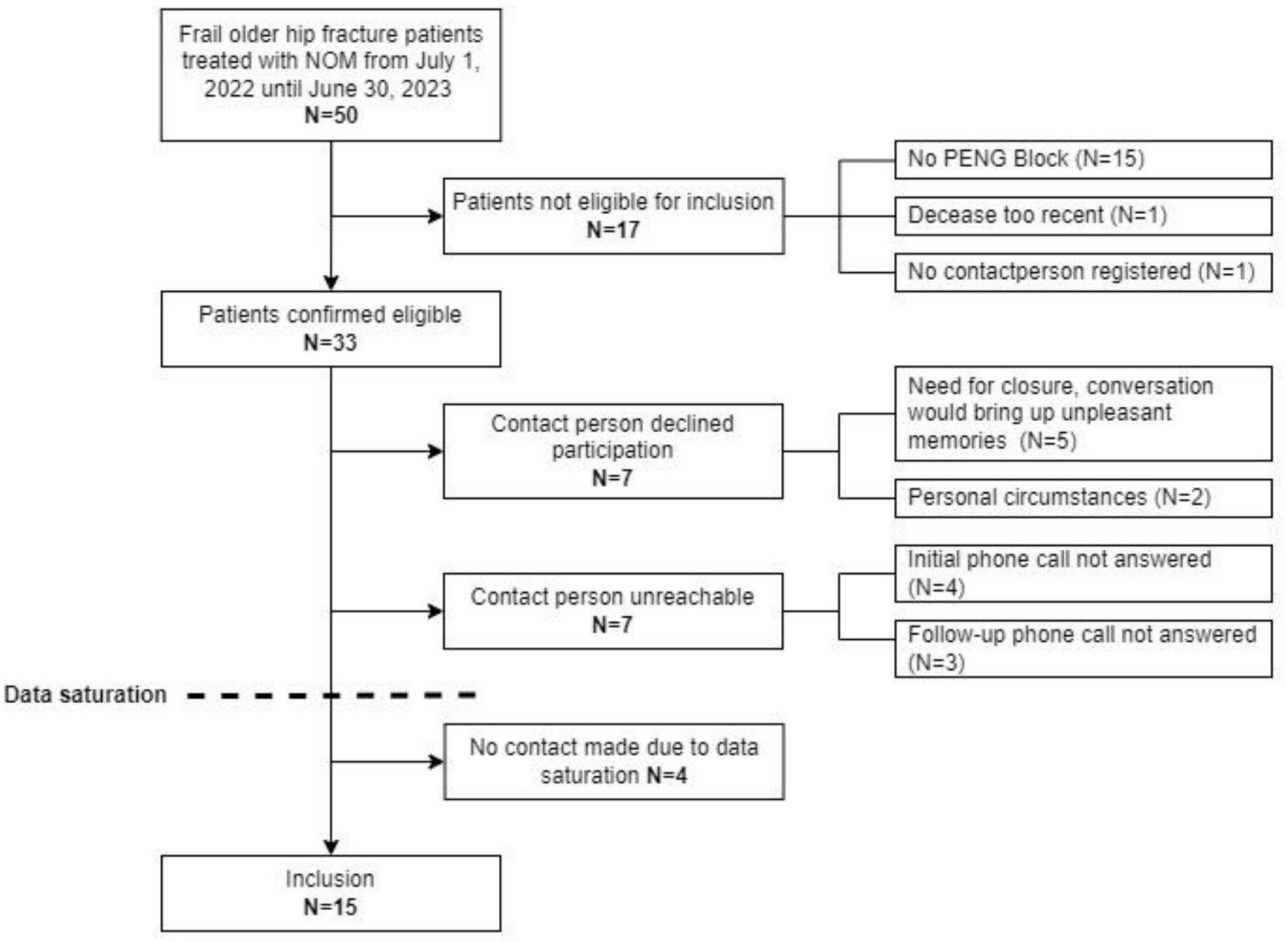
Flowchart participant inclusion. NOM, non-operative management; PENG, PEricapsular Nerve Group.

Convenience sampling was employed in a chronological sequence of patient admission date to mitigate potential volunteer bias. Participants were excluded in case of absent contact details. In the event of a patient’s death, a period of at least 8 weeks from the date of death was maintained to allow time for the acute grieving process in proxies. Unreachable potential participants and reasons to decline participation were recorded. An overview of the recruitment process is provided in figure 1.

Participants were informed of the research protocol and their rights via telephone and could opt to receive written information. They provided oral informed consent for interview participation and permission to use the data for research purposes in an anonymised manner. The Standards for Reporting Qualitative Research guidelines were followed in this report.^29^

### Research methods

The interviews followed a semistructured design. This provided methodological rigour and simultaneously allowed participants and researcher to address additional relevant topics. The interview questions were based on previous research identifying four relevant aspects of treatment experience in NOM: the decision-making process, pain management, patient–relative interaction and the dying process. This is elaborated in online supplemental appendix B.^7 19 30^

Observational data from the electronic patient file included baseline characteristics (ie, sex, age, living situation before event, cognitive function before event, type of fracture, Charlson Comorbidity Index (CCI)) and secondary parameters (ie, time from hip fracture to interview, length of hospital stay, discharge destination, mortality and survival after hip denervation in days). Technical details of the hip denervation procedure were not collected. Data on posthospital care, such as supporting interventions or mobility, were not collected.

### Data collection

The study was conducted by TK, JMvB and HW. TK is a medical doctor with experience in qualitative research. JMvB is a medical doctor and was a final year medical student at the time with limited experience in qualitative research. HW is a geriatric specialist and independent researcher with extensive experience in qualitative research. The observational data were anonymously recorded in the secured electronic data capture system REDCap. TK interviewed the participants by phone. Interview recordings were transcribed ad verbatim by TK and JMvB. Both recordings and transcripts were anonymised and stored on a secure hospital server. After the 12th interview, no novel themes were identified. Three additional interviews were performed to conclude data saturation in agreement between TK and JMvB. Independent researcher HW confirmed the data saturation. The remaining eligible participants were not recruited for this reason.

### Analysis

A thematic discourse analysis was conducted. To systematically analyse and extract themes and discourse from the data, a comprehensive exploration was performed. The thematic analysis adhered to the iterative six-step model for thematic analysis by Braun and Clarke.^28^ In the discourse analysis, the set of 25 tools for the identification of discourses by Gee was employed.^28 31^ After independent familiarisation with the data, TK and HW formulated preliminary themes and discourses. These were confirmed by JMvB. Initial coding of the data was independently performed by TK, JMvB and HW using ATLAS.ti (V.23.1.1.0). Initial intercoder agreement was reached between TK, JMvB and HW, after which the codes were finalised by TK and JMvB. After final intercoder agreement regarding thematic analysis, TK reviewed the transcripts again with a focus on discourse analysis. Intercoder agreement was reached with JMvB and HW regarding relevant discourses. No statistical analyses were performed.

### Patient and public involvement

Patients were not involved in the research, as proxies were approached for study participation. Patients or proxies were not directly involved in the design of this study; however, the research topics were derived from previous qualitative research in a comparable population. Due to the explorative character of the study, participants steered the outcome measures of this study.

## Results

A total of 15 proxies participated in this interview study, as all patients included were either cognitively impaired or deceased. 12 proxies were first-degree relatives to the patient, 2 proxies were second-degree relatives, and 1 proxy was a designated representative without family ties. The patients were mostly women (87%) with a median age of 85 years (IQR 78–90) and exhibiting substantial comorbidity (CCI median 5). All patients required aid in daily activities, and the majority of patients suffered from cognitive decline (67%). Patients stayed in hospital for a median of 3 days (IQR 1–9) and were mostly discharged to a nursing home (67%). The interviews were conducted at a median of 111 days (IQR 96–173) after hip fracture. At the time of the interviews, 13 out of 15 patients had deceased, with a postfracture survival of median 20 days (IQR 12–34). Data on causes of death were unavailable. An overview of patient characteristics is provided in table 1.

**Table 1:** Patient characteristics

	Value
Sample size, n	15
Patient characteristics	
Sex, n (%)	
Male	2 (13)
Female	13 (87)
Median age (IQR) in years	85 (78–90)
Median CCI (IQR)	5 (5–7)
Living situation before fracture, n (%)	
Home, with ADL care	4 (27)
Nursing home	11 (73)
Cognitive impairment before fracture, n (%)	
None	5 (33.3)
Cognitive impairment	2 (13.3)
Dementia	8 (53.3)
Fracture type, n (%)	
Femoral neck fracture	10 (67)
Intertrochanteric fracture	5 (33)
Aftercare characteristics	
Median hospital stay (IQR) in days	3 (1–9)
Discharge location, n (%)	
Home, with ADL care	2 (13)
Nursing home	10 (67)
Hospice	1 (7)
Decease in hospital	2 (13)
Deceased at time of interview, n (%)	13 (87)
Median survival (IQR) after PENG block in days	20 (12–34)
Median time (IQR) from PENG block to interview in days	111 (96–173)

IQR, noted as p25–p75.

ADL, activities of daily living; CCI, Charlson Comorbidity Index; IQR, Inter Quartile Range; PENG, PEricapsular Nerve Group.

**Figure 2:**
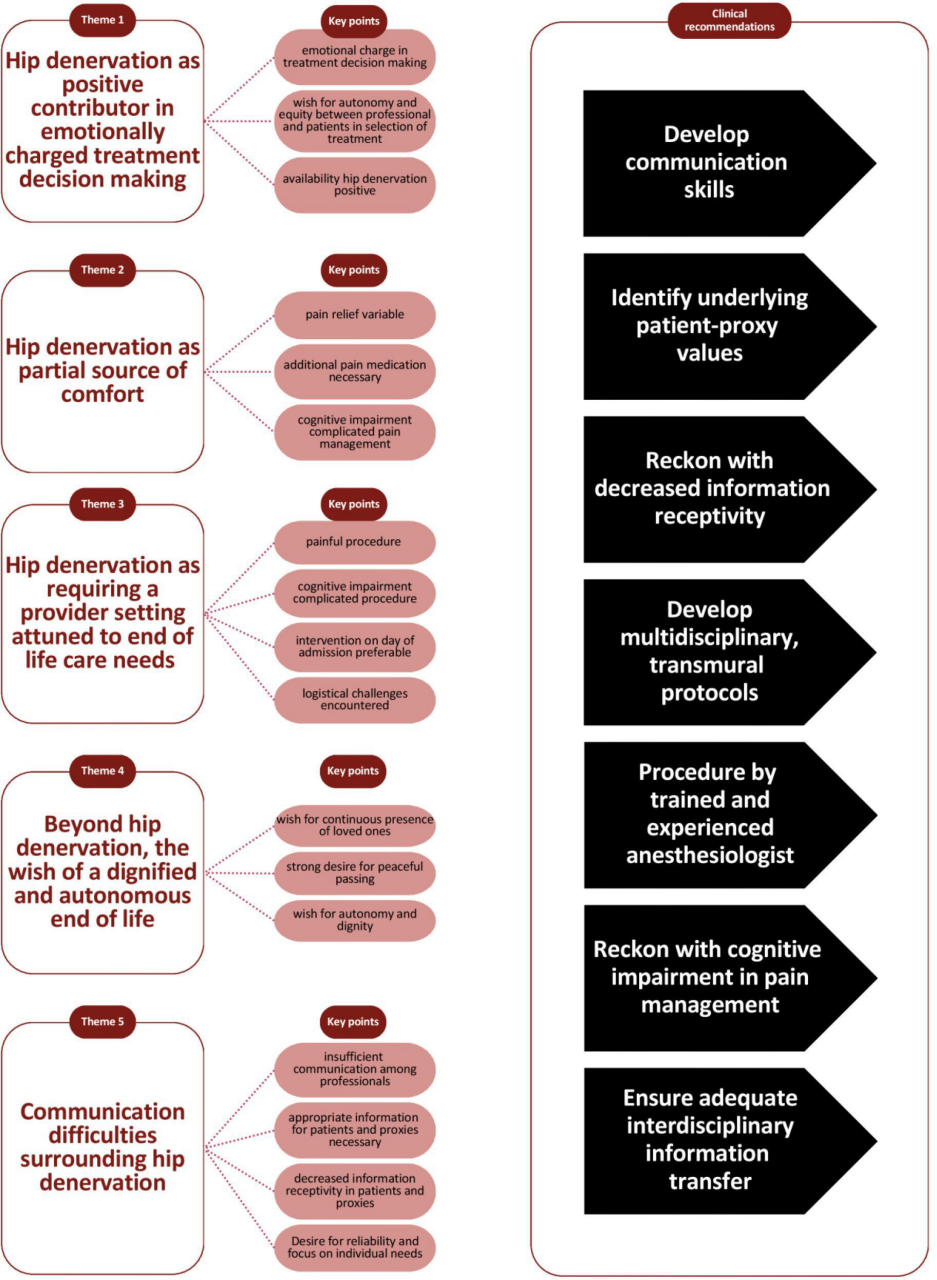
Thematic discourse analysis and clinical recommendations.

The thematic discourse analysis identified five themes with one predominant discourse, which all concerned the broader EOL care setting of hip denervation. The first four themes corresponded with the predetermined topic list. The formation of a fifth theme regarding communication was justified, as communication difficulties were mentioned frequently and with emphasis. The discourse reflected a normative desire for an optimal EOL phase, which was rooted in autonomy, equity, trust, dignity and a tailored approach. The themes with reflecting discourse are described in the following paragraphs with supporting quotes. An overview of themes, key points and recommendations is included in figure 2.

### Theme 1: Hip denervation as a positive contributor in emotionally charged treatment decision-making

The decision-making process between operative and non-operative hip fracture management was emotionally charged. Autonomy and equity between professionals and patients in the selection of treatment were stressed. The availability of hip denervation in NOM was viewed as positive.

#### Emotional charge

The introduction of treatment choice on NOM or OM was referred to as a bad news experience. Hearing about the limited prognosis for very frail patients with a hip fracture was reported as a shock. Participants felt surprised and overwhelmed by both their own emotions and by those of their loved ones.

[About the decision-making process] Well, you were, of course, also overwhelmed by the emotions and such. [Quote No. 1:3]

It was a very, it was one of the most difficult decisions in my life *emotion* (…) Really intense because you actually don't realize it (…) or well, you realize it, but you don't want to believe it, let me put it that way.(…) it was a very difficult choice because I also didn't see it coming at all, so to speak. [Quote No. 7:28]

#### Decision-making inclusion

Participants wanted inclusion in treatment decision-making to determine a course of treatment that was in line with the patient’s wishes. This was described as active engagement of proxies and patients in the decision-making process and adaptation of the decision based on specific patient needs. Participants emphasised the importance and value of a sense of autonomy in ‘*having a choice*’.

I would have liked to hear her decide for herself. That would have been a softer landing, too. (…) You have no choice, I think, but if there was a choice, we would have chosen to follow my mother’s feelings. [Quote No. 15:116 & 15:91]

That was quite nice, actually, that we had the choice at that moment. [Quote No. 10:19]

We had all the explanations there, calmly, adequately, pros and cons (…) discussed with the surgeon and with the nurses (…) and that was very valuable to us. [Quote No. 3:29]

#### Proxy burden

Decision-making usually included consultation with multiple proxies, with possible disagreement among them regarding the optimal course of treatment. Being involved in treatment decision-making and serving as substitute decision-maker for a loved one was reported as ‘*burdensome*’.

You naturally have to decide about the life of another (…) and that is not nice, especially because it is a loved one. [Quote No. 6:8]

#### NOM motivations

Motivations to opt for NOM included advanced age, insufficient added value to quality of life, reduced recovery prospects and alleviating suffering or loneliness. The availability of hip denervation was not a decisive factor in selecting the course of treatment; it was described as ‘*a pleasant surprise*’.

The whole idea behind that PENG block so that they can't feel the pain (…), and let the natural process take its course, I think that’s a very beautiful thing. [Quote No. 1:42]

That at least she wouldn't have pain anymore (…) and that perhaps even if it’s sitting in a wheelchair or occasionally taking a few small steps. [Quote No. 9:42]

### Theme 2: Hip denervation as partial source of comfort

The sufficiency of pain management was a substantial aspect of the participants’ experiences, reflecting a wish for the best possible quality of life. Hip denervation resulted in variable pain relief with a need for additional pain medication. Pain management was more difficult in patients with cognitive impairment.

#### Pain after hip denervation

Variability in the effect of hip denervation included complete, partial or no postprocedural pain relief. Pain increased during movement and care interventions. Additional pain medication included escape medication during mobilisation, or continuous administration via subcutaneous, intravenous or dermal routes. Reported residual pain ranged from none to the extent that daily care was not feasible.

[regarding morphine use] Afterwards, of course, she received the rest of the, uhm, vitamins and (…) Yeah, then she quickly went under, so to speak. [Quote No. 15:107]

If she would just lay in bed it was manageable, but when they would turn her or change her or those kinds of things, or yeah then she had a terrible amount of pain. [Quote No. 3:30]

The pain was definitely very severe, to the extent that she actually didn't want care in the beginning. [Quote No. 12:8]

#### Emotional strain of proxies

Witnessing a loved one endure pain induced emotional strain, and participants expressed concern about accurate pain reporting in patients with cognitive impairment.

Terrible, when you see your mother suffering like that (…) that she had to endure so much pain and only getting worse, we did not expect that. [Quote No. 9:38]

#### Long-term outcome

In the two patients who survived longer than 8 weeks, a varying degree of mild pain was described, controlled with additional pain relief. Mobility returned after 2–3 months, resulting in assisted walking ability or transfers with a passive lift. A wider sitting position was reported. The patient indicated that she *‘has become crooked’*.

You see her just walking and then you don't notice that she’s in pain. She does mention it sometimes, but she still walks and things like that. So yeah, then it’s just fine for us. [Quote No. 4:40]

### Theme 3: Hip denervation as requiring a provider setting attuned to EOL care needs

Hip denervation was performed in the recovery room of the hospital. Movement of patients with cognitive impairment and substantial pain during the procedure was mentioned. Intervention on the day of admission was preferable, as this enabled a quick return home.

#### Logistical challenges

Logistical challenges included the unavailability of a trained anaesthesiologist during weekend hours and difficulties in organising a PENG block in a nursing home setting.

The pain she naturally had from the injection. Yes, that was a little less to say (…) that was terrible (…) a torment for a good cause, so to speak, to block the pain. [Quote No. 15:66]

From Friday to Wednesday she really suffered because they were still going to give her the injection. [Quote No. 8:52]

#### Patient transfer

After hospitalisation, patients were transferred back home, or to a different living environment to receive EOL care. The organisational efforts surrounding this transfer were perceived negatively. Transfer efforts were reported as a ‘*hassle*’, and transfer to the preferred location was not always possible.

She wanted to pass away at home and the organization around it was a mess. [Quote No. 13:4]

I think in hindsight he could have stayed in the hospital. (…) When you think it won't be long anyway. (…) Yeah, I think he would have been better off there. Now you get the feeling like, well, you're written off, then just go home to die. [Quote No. 5:69]

#### Suboptimal posthospital care

Participants expressed dissatisfaction with posthospital care, because of inadequate pain management and mobilisation policies. Participants were confronted with healthcare providers who were not specialised in EOL care for hip fracture patients. Also, heterogeneity in pain management policy among individual healthcare professionals was described.

The physiotherapist would have put my mother right into one of those hammocks, they placed. (…) and I heard her screaming (…) she was in agony, so I demanded that they leave her alone and put her in bed and take care of her well with everything, but not hoist her in such a mat anymore, because that was torture. [Quote No. 6:23 & 6:39]

That was also depending on which nurse was available, because one said yes of course she shouldn't have pain, I'll give her something. The other one said no, she'll get something in an hour. [Quote No. 3:42]

### Theme 4: Beyond hip denervation, the wish of a dignified and autonomous EOL

Treatment experience with hip denervation in the EOL care setting highlights other components besides pain relief. It was characterised by a desire for the continuous presence of loved ones and a strong desire for peaceful passing. This reflected a wish for autonomy and dignity.

#### Proxy presence

Presence of loved ones was described as frequent or continuous due to dependence on care. This presence was not necessarily accompanied by interpersonal interaction. Being present with the patients was described as emotionally burdensome, yet meaningful. Participants expressed added value in being present at the moment of the last breath, so that the patient would not be alone in their last moments. When not present with the last breath, participants expressed that they had to make peace with this by thinking of death as a natural process that cannot be controlled. Presence of family after the last breath was described as a valuable part of the farewell process.

Heavy, but yeah I'm still very glad I did it (…) also knowing that it would be the last days or period of her life, because uhm yeah, it was really made clear to us that yeah she would die here, this won't end well. [Quote No. 3:45]

In such a situation it’s nice that there’s just someone there as soon as she opens her eyes. That I'm just sitting next to her, which gives her a sense of security. That she’s still a bit at home, so to speak. [Quote No. 7:76]

She was able to say goodbye yes (…) on Wednesday evening she grabbed me and started smiling at me. [Quote No. 8:5]

At that moment, we weren't there *emotions* (…) and I found that very difficult, I had been there the whole time and then *cries* (…) at that moment I stood there screaming and I think damn and I think well this is what it is, but I don't believe that anyone consciously chooses it. [Quote No. 3:19]

#### Dignity

During the last phase of life, the experience of dignity was described as positive. The ability to make autonomous decisions and ‘*not being patronised*’ was outlined as a prerequisite for dignity. Also, the presence of laughter and humour from the patient contributed to dignity. Conversely, dignity was compromised by dependence on care or cognitive decline such as failure to recognise loved ones, reliving the past, aggression or motor restlessness.

My father, he still had all sorts of jokes. And he could still talk about everything, about technical things. I could still ask him for advice with a problem with the house or something. (…) In that sense, I think, well, yeah, he passed away in a dignified way rather than completely losing his faculties. He would have found that terrible. [Quote No. 5:13]

She could maintain her dignity, uhm, because she made the decisions and it went as she wanted. [Quote No. 13:30]

#### EOL emotions

Participants reported experiencing uncertainty regarding the prognosis of the patient. A shorter life duration than anticipated elicited disappointment, as participants hoped for improvement and more time for farewells. Conversely, a longer life duration than expected led to a constant feeling of uncertainty. Participants described difficulty and emotions in accepting the EOL, both experienced by the patient and the proxies. Near the EOL, participants observed acceptance and even resignation in patients.

You never know. It could happen any moment. [Quote No. 4:19]

You're sent home with a palliative care plan and you basically assume, well, she won't live much longer. That first weekend was terrible, the first week, the first 10 days, we basically thought, she’s going to die. (…) but the fact is that we're 4 months further and she’s still alive. [Quote No. 1:57]

I would have preferred to talk to her the next day as well. Unfortunately, that didn't happen. (…) she slipped away so quickly. [Quote No. 15:98]

He actually didn't have peace with it, he was really worried. [Quote No. 5:6]

He was actually really worn out. I actually think he held on for so long because he didn't want to leave my mother alone. But uhm yeah for himself I think it was uhm, yeah it was really enough like that. [Quote No. 11:73]

#### Dying process

Dying was described as transcendent with gradual loss of consciousness and breathing ability, where the patient was asleep prior to death. Heavy breathing with attempts to speak was interpreted as a remaining will to live of the patient. The absence of respiratory distress was described as an important feature of a peaceful death.

My mother is very peaceful, very peaceful, that’s how she looked (…) when someone passes away, I wish they would pass away like that (…) because that is super peaceful, not struggling to breathe or anything. [Quote No. 6:68]

She seemed like a kettle, she started bubbling all over, she seemed completely full of mucus and that was very unpleasant to hear (…) that was really terrible. [Quote No. 3:61]

### Theme 5: Communication difficulties surrounding hip denervation

The described difficulties in communication concern clarity, appropriateness and decreased information receptivity for patients and proxies. Clear communication among professionals was mentioned as essential for adequate care delivery. These components reflect a desire for reliability and a focus on individual needs.

#### Information clarity

Clear information without ‘*beating around the bush*’ was valued in interaction with healthcare providers. Simultaneously, the use of definitive language regarding the expected prognosis was perceived as inappropriate, especially when not focused on individual patient or proxy needs.

[When reflecting on communication with treating physicians] we [proxies] have had many really good conversations. Those doctors were really uhm very clear. [Quote No. 4:33]

Your father is just going to die within uhm well a few weeks, a few days. That’s what my father heard. I think you just don't say that so bluntly. [Quote No. 5:25]

#### Limited information receptivity

Participants reported that verbal information was easily forgotten due to the emotional distress and that written information was received, but not read.

In a hurry, you get a brochure [with information on hip denervation] and then you think oh well, I'm not going to read this now. [Quote No. 12:8]

[Regarding information treatment trajectory] you don't absorb it all at the moment they're at the bedside. [Quote No. 15:54]

#### Healthcare provider communication

Communication deficiencies between healthcare providers or institutions were described as highly undesirable. Clear communication and information exchange were mentioned as an important responsibility of healthcare professionals. This reflected a desire for reliability and equity between healthcare providers and patients or proxies.

Everything had gone wrong in the transfer communication (…) that caused all sorts of problems. That wasn't nice. [Quote No. 13:24 & 13:4]

[After the ambulance for transfer arrived 3 hours later than anticipated by hospital personnel] I say, “Finally, are you here?” The man replies, “We can't really predict our arrival time, you know. We're on call and have to respond to accidents, they take priority.” [Quote No. 14:52]

[About variability in expected course of treatment] then you hear they're going to give the injection right away and you're sent home and you have to start all over again on Monday. [Quote No. 8:19]

## Discussion

This interview study explored the treatment experiences with hip denervation via PENG block with phenol after NOM in an EOL care setting. Treatment experience was assessed from the patient–proxy perspective as patients were either deceased or suffered from cognitive impairment.

### Interpretation of key findings

Hip denervation was described as a positive contributor in emotionally charged treatment decision-making around the choice for NOM in the EOL setting. The perception of hip denervation as a positive treatment contributor, yet subsequent to the decision to forgo hip fracture surgery, aligns with previous research. Previous studies emphasised the importance of comprehensive pain management, the decision-making process, patient–relative interaction and the dying process.^7 13 18^ It also highlights the importance of EOL care involvement in very frail hip fracture patients, as this is associated with improved quality of life.^16^ This study shows that the emotional charge of NOM decision-making is also present during the process of hip denervation, possibly influencing the result as these events take place consecutively.

Hip denervation required a provider setting attuned to EOL care needs. The experienced communication difficulties involved decreased information receptivity by patients or proxies and challenges in information transfer among professionals. Previous studies recognise barriers to effective communication in EOL care due to emotional processes.^32^ Physicians delivering care to NOM patients receiving a PENG block may require specific communication skills tailored to the EOL setting. Information exchange among professionals from different organisations is prone to deficiencies, and collaborative standardisation may improve information transfer.^33 34^ In the EOL setting, improvements in communication are crucial in ensuring adequate pain relief.

Hip denervation was reported as a partial source of comfort in this study. This could be explained by the PENG block selectively targeting the innervation of the anterior hip capsule, while sensory innervation of the posterior hip capsule remains intact.^22^,^24^ Moreover, pain is not always solely a result of the fracture. Surrounding tissue may be affected by the trauma or prolonged immobilisation and may be causing pain as well. The effect of the PENG block should be monitored in patients, and supplemented with additional pain management methods if needed. However, the EOL setting requires care that transcends pain relief, focusing on dignity and the presence of loved ones. The underlying discourse reflects a normative perspective on the EOL care setting, where individuals compare the received care with an idealised notion of perfection and controllability. It appears that individuals were caught off guard by distressing news, after which various disappointments occurred throughout the course of treatment. This reveals an underlying discrepancy: an unrealistic picture of patient prognosis, followed by the expectation of a perfect deathbed that sometimes fails to meet up to these expectations. This is accompanied by emotional strain stemming from an unforeseen and definitive event. The identified discourses suggest that the EOL care setting forms the crux in treatment experience in this study, as this surfaced across themes. Although existing EOL care models acknowledge both whole person care and focus on quality of life, no attention has yet been given to the underlying values and care setting as observed in hip fracture care.^14^ This study shows the importance of including underlying values in future EOL care models. The weight of highly unexpected bad news experiences and unfulfilled expectations becomes inherently logical in light of desired values such as reliability, equity, autonomy and preservation of dignity. With identified underlying values, general EOL care models might become more applicable to specific care settings, such as EOL hip fracture care.

### Strengths

This study has several strengths. It is the first study to qualitatively map the experiences with hip denervation with phenol in frail older patients in an EOL care setting. Cognitively impaired patients were included in the study design to enhance generalisability in the frail older hip fracture population.^35^ The study used transparent and widely used qualitative research methods.^28 31^

### Limitations

Limitations of this study included the patient–proxy perspective from which the study was conducted.^26^ Given the EOL care setting and cognitively impaired patients, treatment experience could only be obtained indirectly. This may have induced proxy bias, known to appear in health-related quality-of-life assessments.^36^ Another limitation is the national orientation of the study. Underlying values on EOL care may differ in other cultural contexts. Furthermore, the analysis was conducted from a medical perspective, whereas the outcomes of this study suggest that relevant themes might lie in philosophical or psychological fields. Additionally, due to the explorative design, other themes overshadow the singular experience with hip denervation. Statistical analyses were not conducted due to the qualitative research design.

### Clinical recommendations

Communication difficulties surrounded the hip denervation in frail older hip fracture patients in the EOL phase. Based on this study, clinicians are encouraged to develop clear, reliable and appropriate communication skills in order to create and clarify different possible scenarios in the EOL setting for patients with a hip fracture. It is recommended to identify the underlying values of patients and proxies during consultations, in order to discuss expectations and possibly adjust these to realistic prospects, but also to adjust the treatment plan to these individual values and wishes. The process is emotionally charged, which urges clinicians to reckon with the subsequently decreased information receptivity in patients and proxies. Difficulties due to cognitive impairment should be taken into account during the hip denervation procedure and in the assessment of pain. It is recommended that hip denervation procedures in frail older adults in EOL care are performed by experienced and trained anaesthesiologists. Multidisciplinary treatment protocols that transcend hospital walls are necessary for patients receiving hip denervation in EOL care. Adequate interdisciplinary information transfer should be ensured, because unforeseen imperfections may both negatively influence patient well-being and the psychological processing of proxies.

### Recommendations for future research

This study justifies further research with a focus on the isolated experience with hip denervation or other interventional pain management strategies in patients with a hip fracture. Research in non-EOL settings might provide more insight into the direct patient experience with hip denervation. The results of this study also indicate that additional research on the practical implementation of EOL hip fracture care is needed, to optimise the standardisation of the treatment protocol and enhance interdisciplinary communication.^37^ Involving professionals with experience in EOL care or psychology could benefit the development of protocols and further explorative research in this EOL setting.

## Conclusion

This study highlights the importance of a provider setting attuned to EOL care needs in hip denervation via the PENG block for patients with a hip fracture. In an optimal setting, the procedure is performed as soon as possible, patients and their proxies are provided with clear information, and realistic and adequate information transfer to other professionals is guaranteed. Improved integration of hip denervation in EOL care could be achieved by the formulation of multidisciplinary and transmural protocols. Improvements should be aimed at optimal pain management and effective communication with prioritising personal values.

## Supplementary material

10.1136/bmjopen-2024-095738 online supplemental file 1

10.1136/bmjopen-2024-095738 online supplemental file 2

## Data Availability

Data are available on reasonable request.
