# Supplementary material for: Experience with hip denervation in non-operative hip fracture care for frail older patients in the Netherlands: an interview study
Source: BMJ Open. 2025 May 8;15(5):e095738. doi: 10.1136/bmjopen-2024-095738 (PMC12067832; doi:10.1136/bmjopen-2024-095738)
Supplement: online supplemental file 1 [file bmjopen-15-5-s001.docx]

**APPENDIX A: CLINICAL TREATMENT PROTOCOL PENG BLOCK WITH PHENOL**

**Hospital specific information**

*Purpose:* Clear agreements and procedures regarding pericapsular nerve group (PENG) block in conservative management of hip fracture.

*Scope:* St. Antonius Hospital

*Responsibilities and Authorities:*

- The primary physician is responsible for: Establishing conservative management for hip fracture; Consulting the pain team
- The pain specialist, resident physician, and/or physician assistant in pain medicine are responsible for: Determining the indication for the PENG block; Obtaining informed consent
- The pain consultant is responsible for: Coordinating appointment scheduling; Follow-up consultation if necessary
- The doctor’s assistant is responsible for: Preparing the necessary supplies; Assisting during the treatment; Monitoring the patient after the treatment

**Pre-procedural considerations**

*Indication:* A neurolytic pericapsular nerve group (PENG) block is a treatment applied for pain relief in patients with a hip fracture who are not undergoing surgery.

*Contraindications:* No informed consent; Allergy to local anesthetics or phenol; Note: The use of anticoagulants (even therapeutically) does not constitute a contraindication.

*Advantages:* Low complexity treatment, can be performed quickly; Muscle function remains intact, allowing mobilization; Less morphine required, potentially reducing the risk of delirium

*Risks and Complications:* Bruising at the injection site; Infection (rare); Numbness in part of the thigh; Reduced strength in the leg; Hypersensitivity; Hyper- or dysesthesia after nerve regeneration; Risk of allergic reaction to the anesthetic fluid; Accidental intravenous injection, systemic toxicity leading to heart rhythm disturbances, coma

*Warning:* Note: Phenol is corrosive to the skin and mucous membranes (e.g., eyes).

**Care Process prior to PENG Block**

*Care process by primary physician (ward doctor):*

- Establish conservative management for hip fracture in consultation with the family
- Contact the pain medicine consultant for pain relief request in conservative management of hip fractures. Contact the anesthesiology resident outside office hours.
- Arrange post-treatment care

*Care process by pain team:*

- Determine PENG Block indication
- Obtain informed consent from the patient, or from the first contact person if the patient is unable
- Inform the patient and family about the PENG block treatment
- Schedule the appointment (usually the same day)
- Note: A time-out must be possible with a competent patient; otherwise, it should be in the presence of the first contact person.

**PENG Block procedure**

A neurolytic PENG (pericapsular nerve group) block with phenol is a long-lasting pain block administered by the pain specialist, which disables a large portion of the sensory nerves of the hip. The aim of the block is pain reduction.

*Materials:* Ultrasound device; Ultrasound cover (kit); Ultrasound needles (Braun/Pajunk); Basic set; Phenol 6% 10 ml without contrast; Lidocaine 2%; Ropivacaine 7.5% or Chirocaine 0.5% (used to confirm needle positioning); Sodium chloride 0.9%; 10 ml luer lock syringes; Needles; Safety goggles; Surgical face mask; Sterile gloves; Chlorhexidine 0.5%

*PENG Block procedure:* Performed under ultrasound guidance; Takes approximately 15 minutes; Conducted in the pain management treatment room; Patient is transported to the treatment room in a bed; Pre-procedural fasting is not necessary; IV line as indicated;

**PENG Block Aftercare**

- The effect of the block is expected within 30 minutes, with maximum effect within a day.
- The duration of the block is approximately 6 weeks to 3 months.
- There is usually some residual pain presetnt.
- Mobilization within the bed or to a chair is possible in most patients.
