# Supplementary material for: Experience with hip denervation in non-operative hip fracture care for frail older patients in the Netherlands: an interview study
Source: BMJ Open. 2025 May 8;15(5):e095738. doi: 10.1136/bmjopen-2024-095738 (PMC12067832; doi:10.1136/bmjopen-2024-095738)
Supplement: online supplemental file 2 [file bmjopen-15-5-s002.docx]

**APPENDIX B, INTERVIEW QUESTIONS**

| Theme | Question |
| --- | --- |
| Decision making process | Can you describe the process of making the choice for palliative treatment?  Clarification 1: So how did the choice go?  Clarification 2: So how did you decide? |
| Decision making process | How would you rate the process of making the choice for palliative treatment?  Clarification 1: not the treatment but the making of the decision?  Clarification 2: How did you feel about making the decision? |
| Pain | Can you describe the process since the decision in terms of pain?  Clarification: So how did the pain go during treatment? |
| Pain | How often did ______________ appear to have her/his pain under control? |
| Pain | How would you rate this aspect of ______________'s dying experience? |
| Patient-relative interaction | Can you describe the interaction between ______ and his/her loved ones?  Clarification: How was the contact between ____ and the people around him/her? |
| Patient-relative interaction | How often did ______________ spend time with family and friends? |
| Patient-relative interaction | How would you rate this aspect of ______________'s dying experience? |
| Patient-relative interaction | Was ______________ touched or hugged by her/his loved ones? |
| Patient-relative interaction | How would you rate this aspect of ______________'s dying experience? |
| Dying process | How often did ______________ appear to keep her/his dignity and self-respect? |
| Dying process | How would you rate this aspect of ______________'s dying experience? |
| Patient-relative interaction | Did ______________ say goodbye to loved ones? |
| Patient-relative interaction | How would you rate this aspect of ______________'s dying experience? |
| Dying process | Please describe the dying process? |
| Dying process | How often did ______________ breathe comfortably? |
| Dying process | How would you rate this aspect of ______________'s dying experience? |
| Dying process | How often did ______________ appear to feel at peace with dying? |
| Dying process | How would you rate this aspect of ______________'s dying experience? |
| In depth | Do you think that the patient would choose non operative management with PENG block again if he/she would have to make the decision a second time?  Clarification: how do you look back on the choice for non operative management with PENG block with today's knowledge? |
