# Supplementary material for: Experience with hip denervation in non-operative hip fracture care for frail older patients in the Netherlands: an interview study
Source: BMJ Open. 2025 May 8;15(5):e095738. doi: 10.1136/bmjopen-2024-095738 (PMC12067832; doi:10.1136/bmjopen-2024-095738)
Supplement: Supplementary file 3 [file bmjopen-2024-095738.reviewer_comments.pdf]

PEER REVIEW HISTORY

BMJ Open publishes all reviews undertaken for accepted manuscripts. Reviewers are asked to complete a checklist review form and are provided with free text boxes to elaborate on their assessment. These free text comments are reproduced below.

This paper was submitted to another journal from BMJ but declined for publication following peer review. The authors addressed the reviewers' comments and submitted the revised paper to BMJ Open. The paper was subsequently accepted for publication at BMJ Open.

ARTICLE DETAILS

Title (Provisional)

Experience with Hip Denervation in Non-Operative Hip Fracture Care for Frail Older Patients in the Netherlands: an interview study

Authors

Kroes, Thamar; van Breugel, Johanna M.; Smits, Rachel; Jan Schuijt, Henk; Van der Velde, Detlef; Willems, Hanna; Study Group, Dutch PENG

VERSION 1 - REVIEW

**Reviewer**	**1**
**Name**	**Reviewer 1**
**Affiliation**	
**Date**	**06-Oct-2024**
**COI**	**No competing interest**

Comments not published pending reviewer's consent

**Reviewer**	**2**
**Name**	**Reviewer 2**
**Affiliation**	
**Date**	**06-Oct-2024**
**COI**	**No competing interest**

Comments not published pending reviewer's consent

VERSION 2 - REVIEW

**Reviewer**	**3**
**Name**	**Theodorakis, Nikolaos**
**Affiliation**	**General Hospital of Athens-Amalia Fleming**
**Date**	**05-Jan-2025**
**COI**	**None**

Dear Authors,

Thank you for the opportunity to review this manuscript. Overall, this was an interesting and thought-provoking paper addressing a relevant topic. Below, I provide my detailed comments and suggestions for improvement:

Abstract

* Please try to include more specific key results of the study in the abstract to provide a clearer understanding of the findings.

Introduction

1. Consider starting the introduction with a discussion on population aging, as this provides important context. Incorporating recent studies, such as https://pubmed.ncbi.nlm.nih.gov/39214341/, would strengthen this section.

2. Include a paragraph discussing the challenges and strategies for peri-operative management and decision-making in geriatric patients. The graphical abstract and insights from https://www.mdpi.com/2075-4418/14/19/2153 are particularly relevant here.

3. Clearly outline prior literature on the topic, mentioning any similar studies or reviews. Relevant references include:

* https://pmc.ncbi.nlm.nih.gov/articles/PMC9246498/

* https://pubmed.ncbi.nlm.nih.gov/30985383/

* https://pmc.ncbi.nlm.nih.gov/articles/PMC10342972/.

Methods

* The title "Methods" appears twice as both a heading and sub-heading. Please revise for clarity.

* It would be helpful to report whether any statistical analyses were performed. If no statistical analyses were conducted, explicitly state this in a separate limitations section at the end of the paper, along with other limitations.

Discussion

* Structuring the discussion with sub-headings would improve readability. Suggested sub-sections include:

1. Interpretation of key findings compared to relevant literature.

2. Broader discussion of clinical implications.

3. Identification of gaps in evidence and future research directions.

4. Limitations of the study.

* Please add a paragraph with concise conclusions summarizing the key findings and implications.

Conclusion

* A clear and concise conclusion paragraph summarizing the study's main contributions and clinical relevance would strengthen the manuscript.

I wish the authors the best of luck for success.

**Reviewer**	**4**
**Name**	**Mcliesh, Paul**
**Affiliation**	**The University of Adelaide, Adelaide Nursing School**
**Date**	**15-Mar-2025**
**COI**	**None**

Thank you for the opportunity to review this manuscript.

The paper is long and could be condensed

Abstract

The objective must be re worded to be more obvious to the generalist reader. The notion of 'proxies' is not obvious in this context – consider using an alternate term in the title of the paper

Line 25 expand the abbreviation NOM

Line 24 & 28- I am unsure of what a 'proxy' is in this context- needs explanation

I am assuming relatives, family etc. Is there a reason that the patients were not also included?

I assume some would not have capacity to contribute- dementia etc, but others may. (I acknowledge this is answered in the main body on page 6 Line 82)

Page 4

Line 67 explain the impact of this procedure- ie does nerve function return or is this permanent (aimed to reduce pain until the person death)

Page 7

Line 97 was consent signed and informed?

Were participants informed of their rights (to withdraw etc)

Page 8

Line 131 add year to Braun & Clark citation

Page 10

Line 158 Post # survival days was 20 (Median) Is that short period because other life supporting interventions were withdrawn (ie hydration & nutrition) parenterally, in line with the approach of NOM?

This information helps provide context to the patient situation. Or were there other complications related to limited mobility post fracture – ie PE, chest infection, pressure related injury that shorten the life span?

It would be interesting to note in the proxies were informed of the impact of the NOM and nerve denervation – esp if the patient had been relatively mobile prior to fracture.

It would have been interesting to include proxies of patients who had NOM but without the denervation procedure as well.

The concept of decisions for NOM is influenced by a number of factors- as you have identified. But the risk of proceeding with surgery is e\scalation of co morbidities and risks associated with anaesthetic etc

There is a need to have detail about the types of questions asked in the interviews.

Analysis sections

The reason I make the statement above is that the notion of EOL care decisions and the use of denervation are linked closely and, in the analysis, (process and reporting) it is sometimes hard to see the consideration of the denervation procure and the related implications.

I appreciate it is difficult to separate those 2 concepts (& in realty does not need/should occur) but many of the participants quotes seem to focus primarily on the decision for NOM and the associated implications.

This is partial addressed in 1.4 NOM Motivations – wiudl be useful to discuss this conceptually prior to the analysis section and then follow up in the last section

2.1 & 2.2 Its not clear if the participant quotes re observing pain are based on the patient prior to the denervation or afterwards- if/when it was not that effective at managing the pain- eps on activity such as pressure area care.

These sections need clearer explanations and context

Discussion section

There is room to more closely link the voice of the participants into some of the discussion early in this section.

The issue of how the procedure was communicated to the proxies, the level of detail about its potential effectiveness, the implications of NOM re mobility post discharge etc all warrant more detailed consideration.

There seems to be more of a focus on the themes here, at the expense of what it all means and what is the value of what was found.

Page 23

Line 438-40 this is an important point you raise

Page 24

It would be useful to add titles to this discussion- ie implications for practice/research etc

Some of the focus of this detail is a little generic- its seems to lack an overt link back to the participants voice in the earlier sections

---

VERSION 2 - AUTHOR RESPONSE

Reviewer: 3
Dr. Nikolaos Theodorakis, General Hospital of Athens-Amalia Fleming, National and Kapodistrian University of Athens School of Medicine
Comments to the Author:

1. Dear Authors, Thank you for the opportunity to review this manuscript. Overall, this was an interesting and thought-provoking paper addressing a relevant topic. Below, I provide my detailed comments and suggestions for improvement:
   RESPONSE: Thank you for taking the time to review our manuscript. We have addressed your comments and suggestions for improvement in the manuscript, as elaborated below.

Abstract

2. * Please try to include more specific key results of the study in the abstract to provide a clearer understanding of the findings.
   RESPONSE: Thank you for this remark. We have revised the abstract, the results section now reads as: *'The process surrounding hip denervation was emotionally charged due to the preceding discussion whether or not to operate and the EOL setting. This setting impaired information uptake in participants and hindered communication. Hip denervation was experienced as a partial source of comfort and proxies described the logistics and aftercare as suboptimal. Participants stressed the importance of a dignified and autonomous end of life phase.'*

Introduction

3. Consider starting the introduction with a discussion on population aging, as this provides important context. Incorporating recent studies, such as https://pubmed.ncbi.nlm.nih.gov/39214341/, would strengthen this section.
   RESPONSE: The population aging indeed provides important context to this study. The first sentence of the introduction is therefore dedicated to the aging population and the expected increased. We have altered this sentence and added additional reference to underline the relevance of this context. It now reads as: *"Due to the aging population, hip fractures in frail older patients pose a growing*

healthcare concern". We have added the reference of (Sing et al., 2023) on the expected annual increase of hip fractures in frail older adults.

4. Include a paragraph discussing the challenges and strategies for peri-operative management and decision-making in geriatric patients. The graphical abstract and insights from https://www.mdpi.com/2075-4418/14/19/2153 are particularly relevant here.
   RESPONSE: Thank you for this suggestion, we have included additional emphasis on shared decision making in NOM in the introduction section line 64-64: *'Hip denervation is offered directly after the decision to opt for NOM.'* This is relevant to the experience with PENG block, as the shared decision making surrounding NOM takes place just before the PENG block procedure. This is also something that study participants address.
5. Clearly outline prior literature on the topic, mentioning any similar studies or reviews. Relevant references include:
   * https://pmc.ncbi.nlm.nih.gov/articles/PMC9246498/
   * https://pubmed.ncbi.nlm.nih.gov/30985383/
   * https://pmc.ncbi.nlm.nih.gov/articles/PMC10342972/.

   RESPONSE: Thank you for pointing out these possible relevant references. As the main focus of this study is on acute hip fractures in frail older adults, therefore we have included the reference of Kwun-Tung Ng et al. (2020) and Serra et al. (2023).

Methods

6. * The title "Methods" appears twice as both a heading and sub-heading. Please revise for clarity.
   RESPONSE: Thank you for pointing out this unclarity, we have revised the sub-heading to *"Research methods"*.
7. * It would be helpful to report whether any statistical analyses were performed. If no statistical analyses were conducted, explicitly state this in a separate limitations section at the end of the paper, along with other limitations.
   RESPONSE: Statistical analyses were not performed due to the qualitative research design in which we focused on the depth and diversity in experiences. We elaborated on this in the Methods section line 141-142: *"No statistical analyses were performed."*, and Discussion section line 469-470: *"Statistical analyses were not conducted due to the qualitative research design.".*

Discussion

8. * Structuring the discussion with sub-headings would improve readability. Suggested sub-sections include:
   1. Interpretation of key findings compared to relevant literature.
   2. Broader discussion of clinical implications.
   3. Identification of gaps in evidence and future research directions.
   4. Limitations of the study.

   RESPONSE: We have restructured the discussion section with subheadings and textual improvements.
9. * Please add a paragraph with concise conclusions summarizing the key findings and implications.
   RESPONSE: Thank you for these suggestions, we have improved the discussion with a 'conclusion' paragraph.

Conclusion

10. A clear and concise conclusion paragraph summarizing the study's main contributions and clinical relevance would strengthen the manuscript.
    RESPONSE: We have included a conclusion paragraph at the end of the discussion section.
11. I wish the authors the best of luck for success.
    RESPONSE: Thank you.

Reviewer: 4
Dr. Paul Mcliesh, The University of Adelaide
Comments to the Author:

12. Thank you for the opportunity to review this manuscript.
    RESPONSE: Thank you for taking the time to review our manuscript. We have addressed your reviewer comments in the manuscript, as elaborated below.
13. The paper is long and could be condensed
    RESPONSE: We have reviewed the paper and condensed the text as much as possible.

Abstract

14. The objective must be re worded to be more obvious to the generalist reader. The notion of 'proxies' is not obvious in this context – consider using an alternate term in the title of the paper

RESPONSE: It is indeed important to phrase the objective in a clear way for the generalist reader. We have changed the title of the paper to: *"Experience with Hip Denervation in Non Operative Hip Fracture Care for Frail Older Patients in the Netherlands: an interview study"*. Furthermore, we elaborated on the concept of proxy and the motivation for interviewing proxies instead of patients in the abstract. This is explained in comments 17 and 18 as written below.

15. Line 25: expand the abbreviation NOM
    RESPONSE: Thank you for pointing out this mistake, the abbreviation NOM is eliminated from the abstract. It now reads as: *"Proxies of frail adults aged >70 years with a hip fracture receiving hip denervation via PENG block with phenol in non-operative management between January, 2022 and June, 2023."*
16. Line 24 & 28: I am unsure of what a 'proxy' is in this context- needs explanation. I am assuming relatives, family etc. Is there a reason that the patients were not also included? I assume some would not have capacity to contribute- dementia etc, but others may. (I acknowledge this is answered in the main body on page 6 Line 82)
    RESPONSE: The term 'proxy' may indeed not be familiar to all readers. The rationale for interviewing proxies instead of patients is also relevant for research in frail older adults. We have changed this in the abstract, which now reads as: "*Proxies (first-contact person, first- or second-degree relative) of frail older adults treated between January, 2022 and June, 2023 were interviewed, as patients had either cognitive impairment or deceased*."

Page 4

17. Line 67: explain the impact of this procedure- ie does nerve function return or is this permanent (aimed to reduce pain until the person death)
    RESPONSE: Chemical hip denervation with phenol via PENG block is indeed aimed to reduce pain until the patients' death. We have now elaborated on that in the introduction line 65-66: *"Chemical hip denervation enables long term pain relief aimed to reduce pain until the patients' decease".*

Page 7

18. Line 97: was consent signed and informed? Were participants informed of their rights (to withdraw etc)
    RESPONSE: The participants provided oral informed consent as approved by the Medical Ethics review committee Utrecht (MEC-U). We have clarified this in the methods section line 98-100: *"Participants were informed of their rights and they provided oral informed consent for interview participation and permission to use the data for research purposes in an anonymized manner.'*

Page 8

19. Line 131 add year to Braun & Clark citation
    RESPONSE: We have added the accurate reference to the manuscript.

Page 10

20. Line 158 Post # survival days was 20 (Median) Is that short period because other life supporting interventions were withdrawn (ie hydration & nutrition) parenterally, in line with the approach of NOM? This information helps provide context to the patient situation. Or were there other complications related to limited mobility post fracture – ie PE, chest infection, pressure related injury that shorten the life span?
    RESPONSE: This information could indeed provide context to the patient situation. However, data on post-hospital care, such as the extent of supporting interventions or mobility, were not collected. The causes of death were not available, as patients did not undergo examinations in the EOL setting. We elaborated on this in the manuscript method section line 114-115: *"Data on post-hospital care, such as supporting interventions or mobility, were not collected."* and the results section line 161: *"Data on causes of death were unavailable."*
21. It would be interesting to note in the proxies were informed of the impact of the NOM and nerve denervation – esp if the patient had been relatively mobile prior to fracture.
    RESPONSE: All patients were treated following the hospital treatment protocol available in Appendix A. The treatment was preceded by informed consent with the patient or their proxy. The informed consent includes information on the impact of NOM and nerve denervation. This is now clarified in the methods section line 79-81: *'Hip denervation was preceded by a shared decision making process on NOM and next a consultation by the anesthesiologist for hip denervation. The full clinical treatment protocol is available in Appendix A.'*.
22. It would have been interesting to include proxies of patients who had NOM but without the denervation procedure as well.
    RESPONSE: This would have been interesting indeed. We did not include patients who did not receive PENG block, as denervation with PENG block is applied as standard treatment in our

hospital. Patients who do refrain from PENG block may do so because of very early decease or different fracture characteristics, introducing bias in the study. Furthermore, an interview study in patients not receiving PENG block in NOM treatment was already conducted by Nijdam et al. (2022).

23. The concept of decisions for NOM is influenced by a number of factors- as you have identified. But the risk of proceeding with surgery is e\scalation of co morbidities and risks associated with anaesthetic etc
RESPONSE: We responded to this comment below in comment 27.
24. There is a need to have detail about the types of questions asked in the interviews.
RESPONSE: It is indeed relevant to know the exact questions in this interview study. All interview questions are available in Appendix B. This appendix was previously named 'Interview Topics', which may have introduced confusion. It is now named 'Interview Questions', this is also altered in the methods section line 107-108: *'The interview questions were based on previous research identifying four relevant aspects of treatment experience in NOM'*.

Analysis sections

25. The reason I make the statement above is that the notion of EOL care decisions and the use of denervation are linked closely and, in the analysis, (process and reporting) it is sometimes hard to see the consideration of the denervation procure and the related implications.
I appreciate it is difficult to separate those 2 concepts (& in realty does not need/should occur) but many of the participants quotes seem to focus primarily on the decision for NOM and the associated implications. This is partial addressed in 1.4 NOM Motivations – wiudl be useful to discuss this conceptually prior to the analysis section and then follow up in the last section
RESPONSE: Thank you for this suggestion. These care decisions are indeed consecutive on one another. We have emphasized this in the introduction section: line 64-65 *'Hip denervation is offered directly after the decision to opt for NOM.'*. As well as in the discussion section line 420-422: *'This study shows that the emotional charge of NOM decision making is still present surrounding the process of hip denervation, as these events take place consecutively.'*
26. 2.1 & 2.2 It's not clear if the participant quotes re observing pain are based on the patient prior to the denervation or afterwards- if/when it was not that effective at managing the pain- eps on activity such as pressure area care. These sections need clearer explanations and context.
RESPONSE: Section 2.1 regards the observed pain after hip denervation, the title of the subsection is clarified as follows: *'Pain after Hip Denervation'*. Section 2.2 regards the emotional strain participants feel when observing the pain of their loved ones. The title of the subsection is clarified as follows: *'Emotional strain of proxies'*.

Discussion section

27. There is room to more closely link the voice of the participants into some of the discussion early in this section.
RESPONSE: We have responded to this comment below in comment 33.
28. The issue of how the procedure was communicated to the proxies, the level of detail about its potential effectiveness, the implications of NOM re mobility post discharge etc all warrant more detailed consideration.
RESPONSE: Thank you for pointing this out. We have now elaborated on these issues in de discussion section line 423-427, which reads as: *'Hip denervation required a provider setting attuned to EOL care needs. The experienced communication difficulties concerned decreased information receptivity in patients or proxies, but also the information transfer among professionals. Previous scholars recognize barriers to effective communication in EOL care due to emotional processes [32]. Physicians providing care to NOM patients receiving PENG block, may require specific communication skills for the EOL setting. Information transfer among professionals in different organizations is prone to deficiencies, although collaborative standardization may improve information transfer [33,34]. For patients in the EOL setting, improvements in information transfer are paramount for adequate pain relief. Hip denervation was reported as a partial source of comfort in this study. This is explained by the targeting of the anterior hip capsule, leaving the posterior hip capsule without local anesthesia [22–24]. PENG block effectiveness should be evaluated in patients, and supplemented with other pain relief methods if needed.'*
29. There seems to be more of a focus on the themes here, at the expense of what it all means and what is the value of what was found.
RESPONSE: We have revised the complete discussion section to decrease the emphasis on themes and shift focus to the interpretation and conclusions.

Page 23

30. Line 438-40 this is an important point you raise
RESPONSE: Thank you for the compliment.

Page 24

31. It would be useful to add titles to this discussion- ie implications for practice/research etc
Some of the focus of this detail is a little generic- its seems to lack an overt link back to the participants voice in the earlier sections
RESPONSE: Thank you for these suggestions, we have restructured the discussion sections including subheadings. We have also done several textual revisions to connect the discussion with the research data.

References

Kwun-Tung Ng, T., Chan, W.-S., Peng, P. W. H., Sham, P., Sasaki, S., & Tsui, H.-F. (2020). Chemical Hip Denervation for Inoperable Hip Fracture. *Anesthesia & Analgesia*, *130*(2), 498–504. https://doi.org/10.1213/ANE.0000000000004172

Nijdam, T. M. P., Laane, D. W. P. M., Spierings, J. F., Schuijt, H. J., Smeeing, D. P. J., & van der Velde, D. (2022). Proxy-reported experiences of palliative, non-operative management of geriatric patients after a hip fracture: a qualitative study. *BMJ Open*, *12*(8), e063007. https://doi.org/10.1136/bmjopen-2022-063007

Serra, S., Spampinato, M. D., Riccardi, A., Guarino, M., Fabbri, A., Orsi, L., & De Iaco, F. De. (2023). Pain Management at the End of Life in the Emergency Department: A Narrative Review of the Literature and a Practical Clinical Approach. *Journal of Clinical Medicine*, *12*(13), 4357. https://doi.org/10.3390/jcm12134357

Sing, C.-W., Lin, T., Bartholomew, S., Bell, J. S., Bennett, C., Beyene, K., … Wong, I. C. K. (2023). Global Epidemiology of Hip Fractures: Secular Trends in Incidence Rate, Post-Fracture Treatment, and All-Cause Mortality. *Journal of Bone and Mineral Research*, *38*(8), 1064–1075. https://doi.org/10.1002/jbmr.4821

VERSION 3 - REVIEW

**Reviewer**	**4**
**Name**	**Mcliesh, Paul**
**Affiliation**	**The University of Adelaide, Adelaide Nursing School**
**Date**	**22-Apr-2025**
**COI**	

Thank you for taking time to consider the feedback and make relevant changes.

I am happy with the changes submitted- there are some minor spelling errors and language issues that could be addressed in the proofing process.
